# Thrombotic Storm Induced by Rituximab in a Patient With Pemphigus Vulgaris

**DOI:** 10.7759/cureus.35469

**Published:** 2023-02-25

**Authors:** Abhinav Karan, Amy Kiamos, Pramod Reddy

**Affiliations:** 1 Internal Medicine, University of Florida College of Medicine – Jacksonville, Jacksonville, USA

**Keywords:** pulmonary emboli, deep vein thrombosis (dvt), systemic thrombosis, thrombosis, rituximab, thrombotic storm

## Abstract

Thrombotic storm is a rare hypercoagulable condition characterized by a clinical trigger causing extensive thrombotic events affecting multiple vessels over a short period of time. We present a case of thrombotic storm that developed in a patient who received rituximab therapy. The patient presented to the hospital with dyspnea and shortness of breath and was subsequently diagnosed with extensive thrombotic burden including multiple deep vein thrombi and pulmonary emboli. Hypercoagulable workup for the thrombotic storm was unrevealing with the only identifiable trigger being the rituximab infusion. The patient was treated successfully with anticoagulation and discontinuation of rituximab. There are very few reports highlighting thrombotic events as a complication of rituximab therapy. We aim to increase recognition of thrombotic storm as a potential complication of receiving rituximab therapy.

## Introduction

Thrombotic storm is a rare clinical phenomenon characterized by numerous thrombotic events affecting multiple vascular locations over a short course of time [1]. A single thrombosis can set off a hypercoagulable cascade leading to multiple thrombotic events that can affect both venous and arterial vasculature, commonly involving atypical locations such as a cerebral sinus venous thrombosis. Patients affected by thrombotic storm typically have an underlying hypercoagulable state that is exacerbated by a clinical trigger (i.e., infection, inflammation, pregnancy, and trauma) leading to further thromboembolic events [2,3]. Thrombotic storm is a hematologic emergency that can lead to extensive organ ischemia and death if not appropriately managed. The foundation of treatment is anticoagulation therapy, while other available treatment options include immunomodulatory drugs, surgical thrombectomy, or systemic thrombolysis [1,3]. We present a rare case of a patient with pemphigus vulgaris who developed thrombotic storm after receiving rituximab.

## Case presentation

A 54-year-old female with a past medical history of pemphigus vulgaris compliant with mycophenolic acid 1,500 mg twice a day and prednisone 50 mg once a day therapy presented with a chief complaint of abdominal pain and shortness of breath that started two days prior to presentation. She also endorsed new-onset right lower extremity swelling that began three days prior. She had a recent visit to the emergency department seven weeks prior for an alternate reason where lower extremity doppler ultrasounds were obtained for right calf erythema, which was negative for deep vein thrombi. She is functionally independent at her baseline. She denied any fevers or active skin blistering. Her initial vital signs were significant only for sinus tachycardia to 124 beats per minute.

**Figure 1:**
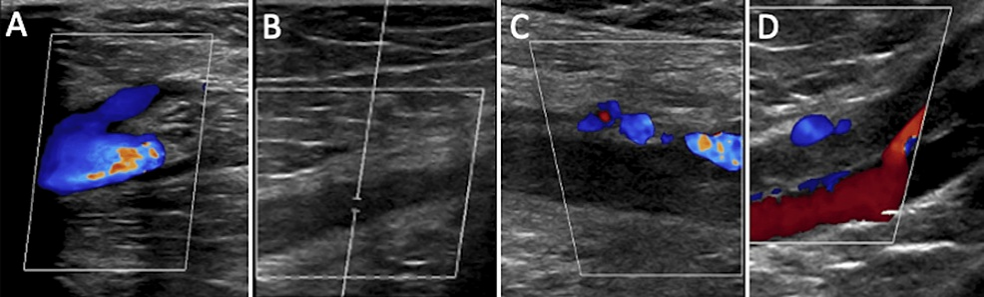
Lower extremity venous doppler ultrasound revealing extensive occlusive bilateral deep vein thrombi involving the right common femoral vein-great saphenous vein junction (A), right femoral vein (B), right popliteal vein (C), and left popliteal vein (D).

**Figure 2:**
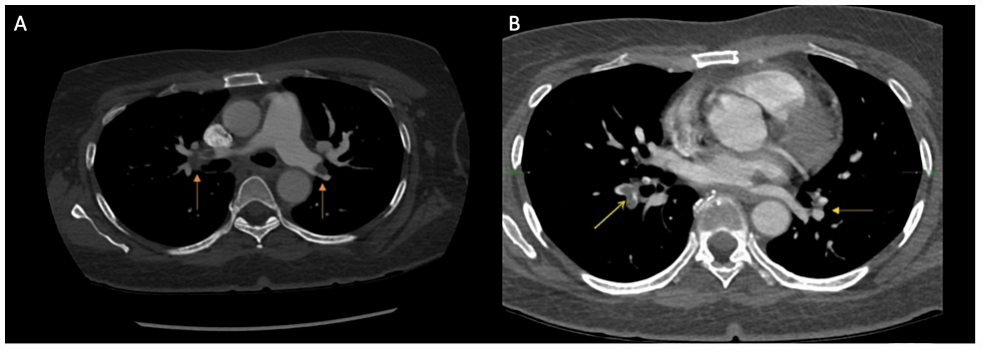
Computed tomographic angiography of pulmonary embolism (CTA PE) revealing a pulmonary filling defect of the right and left main pulmonary arteries (orange arrows) (A) extending into the segmental and subsegmental branches bilaterally (yellow arrows) (B).

**Figure 3:**
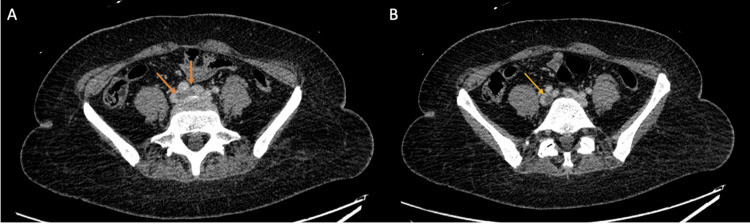
Computed tomography (CT) of the abdomen and pelvis revealing a collapsed inferior vena cava (IVC) with thrombus extending from the bifurcation of the IVC (orange arrows) (A) into the right common iliac (yellow arrow) (B), external iliac, and femoral vein.

Given the concern for a pulmonary embolism (PE), bilateral lower extremity venous doppler ultrasounds and computed tomography pulmonary angiography were performed. The lower extremity ultrasound revealed extensive occlusive bilateral deep vein thrombi, involving the common femoral and popliteal veins of the right lower limb, and popliteal vein on the left lower extremity (Figure 1). Her computed tomographic angiography of pulmonary embolism (CTA PE) revealed a pulmonary filling defect of the bilateral main pulmonary arteries, right greater than left, extending into segmental and subsegmental branches bilaterally with no evidence of right heart strain (Figure 2). A subsequent CTA of the abdomen and pelvis revealed a collapsed inferior vena cava (IVC) with a thrombus extending from the bifurcation of the IVC into the right common iliac, external iliac, and femoral vein (Figure 3).

Despite the extensive thrombosis, she remained hemodynamically stable and did not require any emergent intervention. The pulmonary embolism severity index score was 49 indicating a low-risk PE with 0-1.6% 30-day mortality. Transthoracic echocardiogram (TTE) showed a mildly enlarged right ventricle (RV) with normal RV systolic function and a left ventricle ejection fraction of 50-55%. The high-sensitivity troponin levels were <6 ng/L (reference range: <14 ng/L), and the N-terminal pro B-type natriuretic peptide (NT-proBNP) was 58 pg/ml (reference range: 0-125 pg/ml). Several possible etiologies for the extensive clot burden were considered. She was up to date on her cancer screening and had no evidence of malignancy in her imaging studies. Her complete blood count and comprehensive metabolic panel were normal with no evidence of hepatic or renal dysfunction. She was not using any oral contraceptive pills. Her international normalized ratio (INR), prothrombin time (PT), and activated partial thromboplastin time (aPTT) were unremarkable at 1.1, 12.2, and 28 seconds, respectively (INR reference range: 0.8-1.1s; PT reference range: 25-37s; aPTT reference range: 25-37s). Urine studies were unremarkable and revealed no evidence of nephrotic range proteinuria. Inpatient autoimmune tests with ANA, c-ANCA, p-ANCA, RF, anti-CCP, anti-SSa, and anti-SSb were all negative. She received a comprehensive workup for hypercoagulability prior to the initiation of anticoagulation including Factor V Leiden, Prothrombin mutation 20210, antithrombin III, protein C, and protein S, all of which were negative. Testing for antiphospholipid antibody syndrome was also unremarkable with negative cardiolipin antibodies and beta-2-glycoprotein titers. Her COVID polymerase chain reaction testing was also negative.

After extensive workup for an etiologically obscure thrombotic storm, she endorsed that she recently received an infusion of rituximab two weeks prior to her current presentation. Her rituximab therapy was subsequently discontinued, and the patient was started on systemic anticoagulation. She has remained compliant with therapy and with no further complications.

## Discussion

A thrombotic storm is a very infrequently reported phenomenon described in the literature as multiple thromboembolic events occurring in multiple anatomical locations over a short period of time [3]. Our patient presented with an extensive thrombotic burden in the setting of recent rituximab infusion with no other identifiable etiology. Her hypercoagulability workups were all negative. This patient had pre-existing risk factors for venous thromboembolism (VTE), including a history of pemphigus vulgaris, glucocorticoid use, and mycophenolic acid use. However, the patient had been on glucocorticoid and mycophenolic acid therapy for several years and had well-controlled pemphigus vulgaris making a dramatic presentation with a thrombotic storm unlikely. The temporal relationship between the initiation of rituximab and the onset of her extensive venous thrombi is established in our case, particularly given that her prior imaging was negative for any thrombi.

Rituximab is a rarely described etiology of VTE. It has been described as occurring more frequently in patients with immune thrombocytopenia and systemic lupus erythematosus receiving rituximab than in the general population [4,5]. Additionally, there are very few case reports documenting isolated thrombotic complications of rituximab therapy, including literature establishing a higher incidence of VTE in patients with pemphigus vulgaris [6]. Given the temporal relationship between initiating rituximab and the onset of the thrombotic storm, in addition to established literature, it is prudent for physicians to be aware of this complication of rituximab therapy, particularly as a biologic therapy for autoimmune disease increases.

## Conclusions

This case highlights a rare presentation of a patient developing thrombotic storm induced by rituximab therapy. We aim to increase awareness of the unusual relationship between thrombotic storm and rituximab. It is important for providers to recognize thrombotic storm as a potential complication of rituximab therapy to guide appropriate therapy and prevent devastating outcomes.
